# Identification and validation of immunogenic cell death-related score in uveal melanoma to improve prediction of prognosis and response to immunotherapy

**DOI:** 10.18632/aging.204680

**Published:** 2023-05-03

**Authors:** Xiaoyan Li, Jing Kang, Jing Yue, Dawei Xu, Chunhua Liao, Huina Zhang, Jin Zhao, Qiongwen Liu, Jinke Jiao, Lin Wang, Guoyin Li

**Affiliations:** 1 Department of Central Laboratory, Shanxi Provincial People’s Hospital, Taiyuan, Shanxi, China; 2 Department of Blood Transfusion, Shanxi Provincial People’s Hospital, Taiyuan, Shanxi, China; 3 Department of Clinical Laboratory, Shanxi Bethune Hospital, Shanxi Academy of Medical Sciences, Tongji Shanxi Hospital, Third Hospital of Shanxi Medical University, Taiyuan, Shanxi, China; 4 Department of Physiotherapy and Rehabilitation, The Second Affiliated Hospital of Air Force Military Medical University, Xi'an, Shaanxi, China; 5 College of Life Science and Agronomy, Zhoukou Normal University, Zhoukou, Henan, China; 6 Department of Geriatrics, Xijing Hospital, The Air Force Military Medical University, Xi'an, Shaanxi, China; 7 Key Laboratory of Modern Teaching Technology, Ministry of Education, Shaanxi Normal University, Xi’an, Shaanxi, China; 8 Academy of Medical Science, Zhengzhou University, Zhengzhou, Henan, China

**Keywords:** uveal melanoma, immunogenic cell death, machine learning, prognostic model, immunotherapy

## Abstract

Background: Immunogenic cell death (ICD) could activate innate and adaptive immune response. In this work, we aimed to develop an ICD-related signature in uveal melanoma (UVM) patients and facilitate assessment of their prognosis and immunotherapy.

Methods: A set of machine learning methods, including non-negative matrix factorization (NMF) method and least absolute shrinkage and selection operator (LASSO) logistic regression model, and bioinformatics analytic tools were integrated to construct an ICD-related risk score (ICDscore). CIBERSORT and ESTIMATE algorithms were used to evaluate the infiltration of immune cells. The Genomics of Drug Sensitivity in Cancer (GDSC), cellMiner and tumor immune dysfunction and exclusion (TIDE) databases were used for therapy sensitivity analyses. The predictive performance between ICDscore with other mRNA signatures was also compared.

Results: The ICDscore could predict the prognosis of UVM patients in both the training and four validating cohorts. The ICDscore outperformed 19 previously published signatures. Patients with high ICDscore exhibited a substantial increase in immune cell infiltration and expression of immune checkpoint inhibitor-related genes, leading to a higher response rate to immunotherapy. Furthermore, the downregulation of poly (ADP-ribose) polymerase family member 8 (PARP8), a critical gene involved in the development of the ICDscore, resulted in decreased cell proliferation and slower migration of UVM cells.

Conclusion: In conclusion, we developed a robust and powerful ICD-related signature for evaluating the prognosis and benefits of immunotherapy that could serve as a promising tool to guide decision-making and surveillance for UVM patients.

## INTRODUCTION

Immunotherapy has revolutionized anti-tumor treatment, particularly for cancer patients at an advanced stage. Immune checkpoint inhibitor (ICI) based therapeutic strategy has been the standard first-line anti-cancer treatment for patients with advanced stages of cutaneous melanoma (SKCM) [1], non-small-cell lung cancer (NSCLC) [2, 3], hepatocellular carcinoma (HCC) [4], and esophageal cell squamous carcinoma (ESCC) [5]. In addition, recent efforts are also diverting to exploiting immunotherapy in patients at an early stage in the neoadjuvant therapy, leading to some promising results [6, 7]. Despite this significant advance, some tumors, such as uveal melanoma (UVM) [8] and pancreatic cancer [9], show minimal or no sensitivity to immunotherapy.

Why do UVM patients respond poorly to ICIs? Some researchers proposed that although UVM and SKCM have a common origin from neural crest-derived cells, UVM patients have a lower tumor mutational burden (TMB), which is fundamental in synthesizing neoantigen and correlates with the response to immunotherapy [10, 11]. Besides, UVM cells might synthesize and secrete vascular endothelial growth factor (VEGF) and basic fibroblast growth factor (bFGF), leading to vascular abnormalities and facilitating immune evasion [12–14]. In addition, a higher ratio of exhausted CD8+ T cells observed in UVM patients might also contribute to the unsuccessful immunotherapy in this disease and shorter overall survival of these patients [15, 16].

The exact mechanism of this low responsive rate to immunotherapy is complex. Besides, tumor heterogeneity generally referred as genetic and phenotypic difference, has a profound impact on the biological behaviors of the tumor, therapeutic sensitivity, and prognosis of cancer patients [17]. Consequently, effective biomarkers to guide clinical decision-making in cancer treatment would be helpful. For example, the expression of PD-L1 has been used in clinics to guide the treatment of NSCLC patients at an advanced stage [18]. Unfortunately, the expression of PD-L1 seems less valuable in the treatment guideline for other types of cancer [19, 20]. To find useful biomarkers, researchers have constructed mRNA-related signatures promising in predicting prognosis and response to immunotherapy [21–23]. Immunogenic cell death (ICD) is a kind of regulated cell death (RCD) that could trigger antigen-specific adaptive immunological responses [24]. Due to the tight association between ICD and the immune system, induction of ICD becomes a strategy in designing anti-cancer agents. Besides, some studies also developed ICD-related prognostic models in some types of cancer, which seemed helpful in predicting immunotherapy response [25–27].

The role of ICD in UVM has been rarely investigated, and whether ICD-related genes could be used to classify UVM patients and guide anti-cancer treatment is still unclear. In this work, we developed a novel ICD-related risk score (ICDscore) in UVM by integrating several machine learning methods and five independent UVM cohorts and combining it with bulk RNA-seq data and clinical information. More importantly, we compared ICDscore with 19 previously published mRNA signatures and with clinical parameters in this disease. The ICDscore might be a useful in predicting prognosis and selecting UVM patients for immunotherapy.

## MATERIALS AND METHODS

### Public data acquisition and processing

The RNA sequence data and clinical information of the TCGA-UVM cohort (*n* = 80) were obtained from the Cancer Genome Atlas (TCGA) database (https://portal.gdc.cancer.gov). The GSE22138 (*n* = 63), GSE84976 (*n* = 28), GSE44295 (*n* = 57), and GSE39717 (*n* = 31) were obtained from the Gene Expression Omnibus (GEO) database (https://ncbi.nlm.nih.gov/gds). All of the datasets were processed as described in our previous study [28]. Since all data sets used in this study were downloaded from public databases, an extra ethical approval was not necessary.

### Cell culture and treatment

Human uveal melanoma cell MUM2B and C918 were obtained from Cell Bank of Shanghai Institute for Biological Sciences, Chinese Academy of Sciences. Cells were cultured in DMEM medium, containing 10% FBS, and maintained in an incubator with constant temperature and CO2. The use of uveal melanoma cells was approved by the Ethics Committee of Shanxi Provincial People's Hospital (2021-196).

### Non-negative matrix factorization (NMF) clustering

NMF clustering was conducted by using the “NMF” package in R, based on the gene expression of ICD related genes (REF). The ranks were set from 2 to 10 to do the NMF rank survey. The optimal molecular subtypes were determined according to the cophenetic coefficient and the consensus matrix.

### Construction of ICDscore in UM

The NMF clustering method was used to classify UM patients into two clusters (C1 and C2), based on the gene expression of ICD related genes. The differentially expressed genes (DEGs) between C1 and C2 were subsequently obtained by the “limma” package in R (REF). These DEGs were analyzed by univariate Cox regression, and those genes with a significant prognostic value (*p* < 0.1) in the TCGA-UVM, GSE22138, and GSE84976 cohorts. Subsequently, a total of 104 DEGs were then input into a Least absolute shrinkage and selection operator (LASSO) regression model in TCGA-UVM cohort and 10 genes were screened out. These genes were further input into a stepwise Cox regression model (bidirectional elimination), and the analyses generated 5 key genes and their corresponding coefficients. The risk score for each patient was calculated by the following formula:


$$score=\sum_{i}Coefficient\text{(}Gene\;i\text{)}\times Expression\text{ (}Gene\;i\text{)}$$

The ICDscore of patients in each cohort was calculated with the formula: ICDscore = (score-Min)/absolute (Max), as reported in our previous studies [21, 28].

### Immune profile analysis

The infiltration ratio of 22 immune cells in patients was calculated by the CIBERSORT algorithm in R software (REF), as reported in our previous study [28]. The ImmuneScore and StromalScore of each patient were calculated by the ‘estimate’ package in R [29].

### Enrichment analysis

Gene Set Enrichment Analysis (GSEA) of SKCM patients was performed by the ‘clusterProfiler’ package in R. The *c5.go.bp.v2022.1.Hs.symbols.gmt* was chosen as the gene set database. The ‘GseaVis’ package in R was used for visualization [30].

### Cell transfection and qRT-PCR

The siPARP8 and negative control sequences were purchased from Shanghai Gemma Gene. Lipo8000™ (Beyotime, C0533, China) transfection reagent was used to transfect siRNAs into cells. The qPCR was performed to detect the expression levels of target genes by BeyoFast™ SYBR Green qPCR Mix (Beyotime, D7262, China). The sequences of siRNA and qPCR primer are shown in Supplementary Table 1.

### Cell counting kit-8 (CCK-8) assay

C918 and MUM2B cells were placed in 96-well plates and detected at 0, 24, 48, and 72 h respectively as described in our previous study [31]. The CCK-8 kit (Beyotime, C0038, China) was used to incubate cells, and the absorbance value of cells at 450 nm was detected by enzyme labeling instrument (Biorad 680, USA).

### Cell scratch test

The cells were plated in 6-well plates, scratched with a 200 μL pipette gun. After that, the cells were cultured in the medium containing 1% FBS, and photos were taken at 0 and 24 hours respectively.

### Statistical analysis

All the data were processed, analyzed and visualized by R software (version 4.1.3). In addition to the packages mentioned above, other packages in R used in this work included “tidyverse”, “survival”, “msigdbr”, “dplyr”, “org.Hs.eg.db”, “ggplot2”, “glmnet”, “scales”, “aplot”, “survivalROC”, “ggrepel”, “enrichplot”, “corrplot”, “survminer”, “timeROC”, “rms”, “pec”, “ggalluvial”, “VennDiagram”, “ggh4x”, “patchwork”, “Oncopredict”, and “CompareC”. The Kaplan-Meier method was used for prognosis analyses. The Correlation analyses were conducted with the Pearson method. The comparison of categorical variables between two groups was conducted with the chi-squared *t*-test. The continuous variables were compared with the Wilcoxon rank-sum test. A value of *p* < 0.05 was considered to be statistically significant (^*^p < 0.05; ^**^p < 0.01; ^***^p < 0.001; ^****^p < 0.0001).

## RESULTS

### Unsupervised clustering of ICD-related genes in uveal melanoma

**Figure 1:**
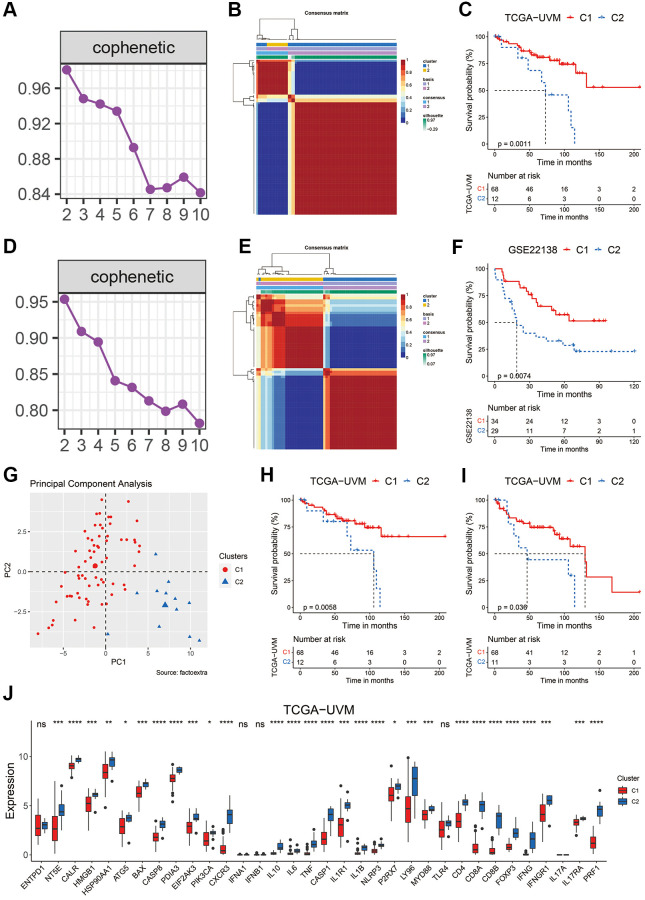
**Clustering of uveal melanoma patients based on ICD-related genes.** (**A**, **B**) The cophenetic coefficient map (**A**) and heatmap (**B**) of NMF clustering results of UVM patients from the TCGA-UVM cohort. (**C**) Kaplan-Meier curves of OS in the C1 and C2 clusters of UVM patients from the TCGA-UVM cohort. (**D**, **E**) The cophenetic coefficient map (**D**) and heatmap (**E**) of NMF clustering results of UVM patients from the GSE22138 cohort. (**F**) Kaplan-Meier curves of OS in the C1 and C2 clusters of UVM patients from the GSE22138 cohort. (**G**) PCA analysis of the C1 and C2 cluster of UVM patients from the TCGA-UVM cohort. (**H**, **I**) Kaplan-Meier curves of DSS (**H**) and PFI (**I**) in the C1 and C2 clusters of UVM patients from the TCGA-UVM cohort. (**J**) The expression levels of the ICD-related genes in C1 and C2 clusters of UVM patients from the TCGA-UVM cohort. Abbreviation: Ns: not significant. ^*^p < 0.05; ^**^p < 0.01; ^***^p < 0.001; ^****^p < 0.0001.

To explore the potential value of ICD-related genes in uveal melanoma, the nonnegative matrix factorization (NMF) consensus clustering was performed based on the expression values of the ICD-related genes in the TCGA-UVM cohort. According to the cophenetic coefficient and the consensus matrix (Figure 1A, 1B), the uveal melanoma patients were divided into two clusters. Figure 1C shows that 68 patients were distributed to the C1 cluster and had significantly longer overall survival (OS, *p* = 0.0011) than those in the C2 cluster (*n* = 12). A similar procedure was performed using the GSE22138 cohort. Patients in this cohort were also divided into two clusters, with patients in the C1 cluster (*n* = 34) showing a significantly prolonged OS than those in the C2 cluster (*n* = 29) (Figure 1D–1F). PCA analysis in the TCGA-UVM cohort further indicated that the C1 and C2 clusters had different distributions (Figure 1G). In addition, those UVM patients in the C1 cluster also showed a significantly longer disease specific survival (DSS, Figure 1H, *p* = 0.0058) and progression-free interval (PFI, Figure 1I, *p* = 0.038). Interestingly, the patients in the C1 cluster had significantly lower expression of most of the ICD-related genes (Figure 1J). These preliminary results suggested that ICD-related genes might have certain impact on the development and advancement of UVM.

### Development of ICD-related signature in uveal melanoma

**Figure 2:**
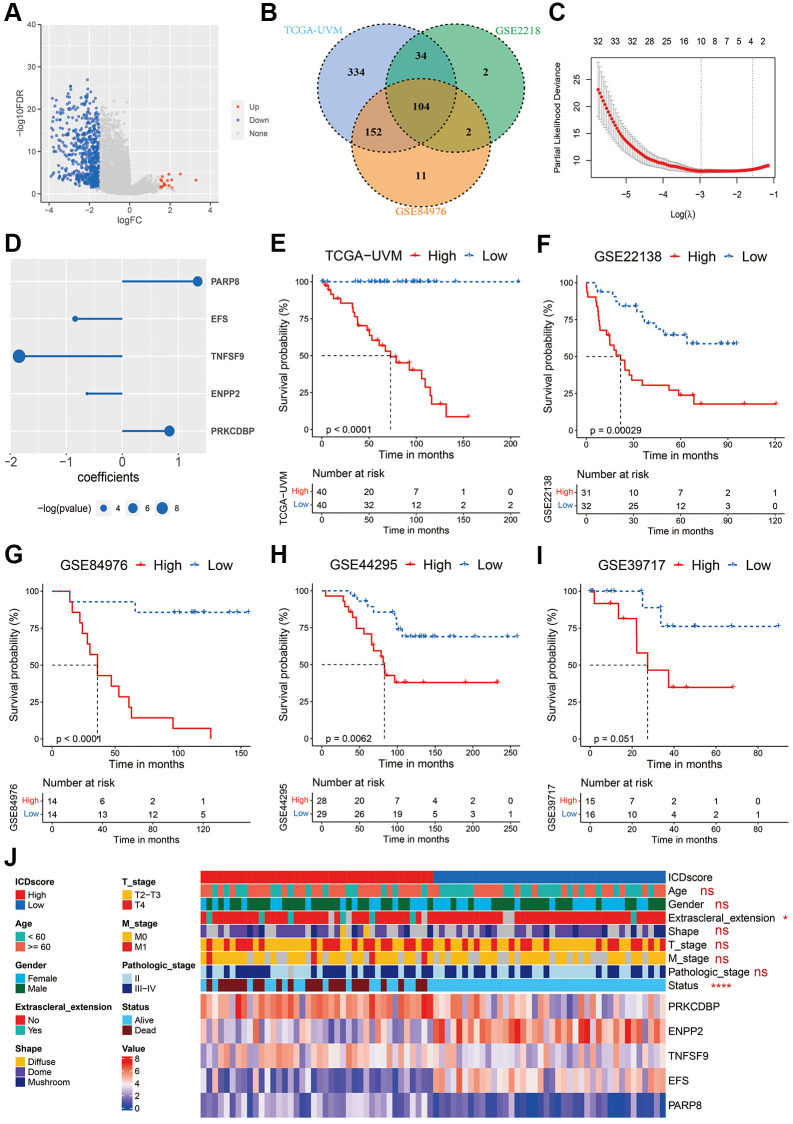
**Construction of the ICDscore.** (**A**) The volcano plot of DEGs between the C1 and C2 clusters of UVM patients from the TCGA-UVM cohort. (**B**) Venn diagram of DEGs with significant prognostic *p*-value < 0.1 in the TCGA-UVM, GSE2218 and GSE84976 cohorts. (**C**) The LASSO Cox regression model was constructed from 104 common DEGs, and 10 core genes selected according to the best fit profile. (**D**) Multi-variate Cox analysis revealed that five genes were independent prognostic factors for UVM patients from the TCGA-UVM cohort. (**E**–**I**) Kaplan-Meier curves of OS in UVM patients from high-ICDscore and low-ICDscore subclusters of TCGA-UVM (**E**), GSE22138 (**F**), GSE84976 (**G**), GSE44295 (**G**) and GSE39717 (**I**) cohorts. (**J**) Clinical features and RNA expression level of five core genes in patients from high-ICDscore and low-ICDscore subclusters of the TCGA-UVM cohort. Abbreviation: Ns: not significant. ^*^p < 0.05; ^**^p < 0.01; ^***^p < 0.001; ^****^p < 0.0001.

To construct an ICD-related signature that helps to recognize UVM patients with different prognoses, we first analyzed the differentially expressed genes (DEGs) between patients in C1 and those in C2 clusters. As shown in Figure 2A, a total of 675 DEGs were identified in the TCGA-UVM cohort with a logFC (fold change) ≥ 1.5 and adjusted *p*-value < 0.05 (Supplementary Table 2). Subsequently, these DEGs underwent univariate Cox analyses in the three independent cohorts: TCGA-UVM, GSE22138, and GSE84976. 104 common DEGs were found to show a significant *p*-value < 0.1 across the cohorts (Figure 2B). A two-step procedure was performed to select key genes that could differentiate between C1 and C2 clusters. Firstly, the 104 common DEGs were analyzed by a LASSO Cox regression model using the TCGA-UVM cohort as in our previous studies [21, 28, 32]. Based on the optimal value of λ (Figure 2C), the following ten genes were identified as significant: S100 calcium binding protein A4 (S100A4), CD79B, protein kinase C delta binding protein (PRKCDBP, also named as caveolae associated protein 3 (CAVIN3)), ectonucleotide pyrophosphatase/phosphodiesterase 2 (ENPP2), TNF superfamily member 9 (TNFSF9), embryonal Fyn-associated substrate (EFS), megakaryocyte-associated tyrosine kinase (MATK), nuclear factor of activated T cells 4 (NFATC4), interferon stimulated exonuclease gene 20 (ISG20), and the poly(ADP-ribose) polymerase family member 8 (PARP8). Secondly, these ten genes were further analyzed in a step-wise Cox regression model using the TCGA-UVM cohort obtaining the following candidates: PRKCDBP, ENPP2, TNFSF9, EFS, and PARP8. Multi-variate Cox analysis revealed that all these five genes were an independent prognostic factor for UVM (Figure 2D). The ICDscore of each sample was calculated based on the transcriptional profiles and coefficients of these five genes, as described in the Method section. In all cohorts, the UVM patients were divided high- and low-ICDscore subgroups by setting the median value of the ICDscore in each cohort as cutoff. In the training dataset (TCGA-UVM), patients in the low-ICDscore subgroup showed a significantly prolonged median OS than that in the high-ICDscore subgroup (Figure 2E, not reached vs. 72.75 months, *p* < 0.0001). In external validating cohorts, patients with low-ICDscore also exhibited significantly longer survival time than those with high-ICDscore in the GSE22138 (Figure 2F, *p* = 0.00029), GSE84976 (Figure 2G, *p* < 0.0001), and GSE44295 cohorts (Figure 2H, *p* = 0.0062). In the GSE39717, although the result did not reach statistical significance (Figure 2I, *p* = 0.051), patients with high-ICDscore apparently had shorter median metastasis free survival (MFS). Besides, ICDscore-based stratification had no statistical relevance with most clinical features, such as age, gender, shape of tumor, T stage, M stage and pathological stage (Figure 2J). But the high-ICDscore subgroup had a significantly higher percentage of melanoma patients with extrascleral extension (*p* < 0.05) and dead status (*p* < 0.0001).

**Figure 3:**
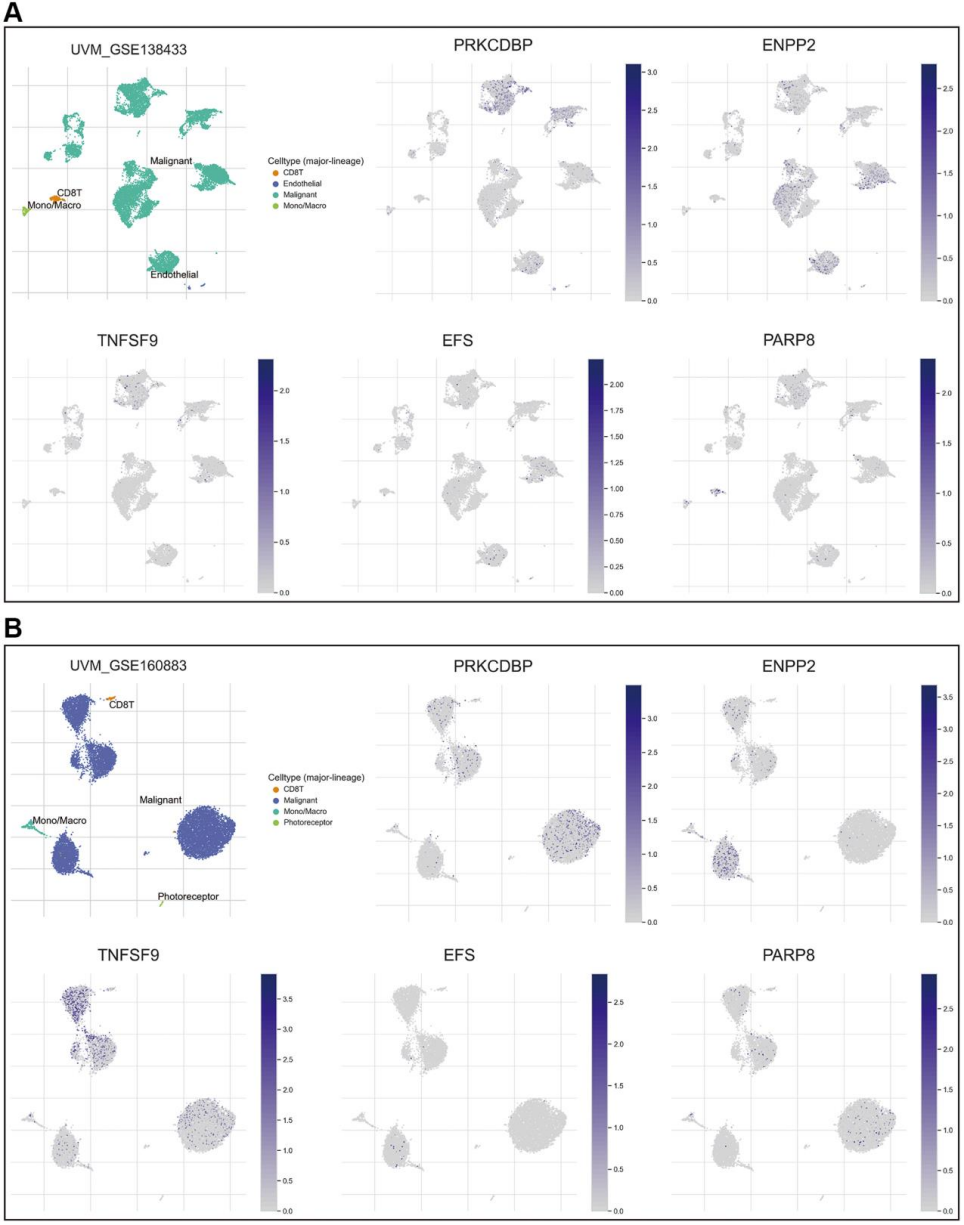
**Expression pattern of the 5 crucial genes.** (**A**, **B**) The expression pattern of the five crucial genes in the GSE138433 (**A**) and GSE160883 (**B**) cohorts. Abbreviation: Ns: not significant. ^*^p < 0.05; ^**^p < 0.01; ^***^p < 0.001; ^****^p < 0.0001.

To further explore the expression pattern of these five key genes, single-cell RNA sequence analyses were performed. As shown in Figure 3A, 3B, all the five crucial genes were predominantly expressed in the malignant cells. Besides, PARP8 could also be detected in CD8 T cells (Figure 3A) and TNFSF9 could also be detected in monocytes and macrophages (Figure 3B).

### Evaluation of the ICDscore in UVM

**Figure 4:**
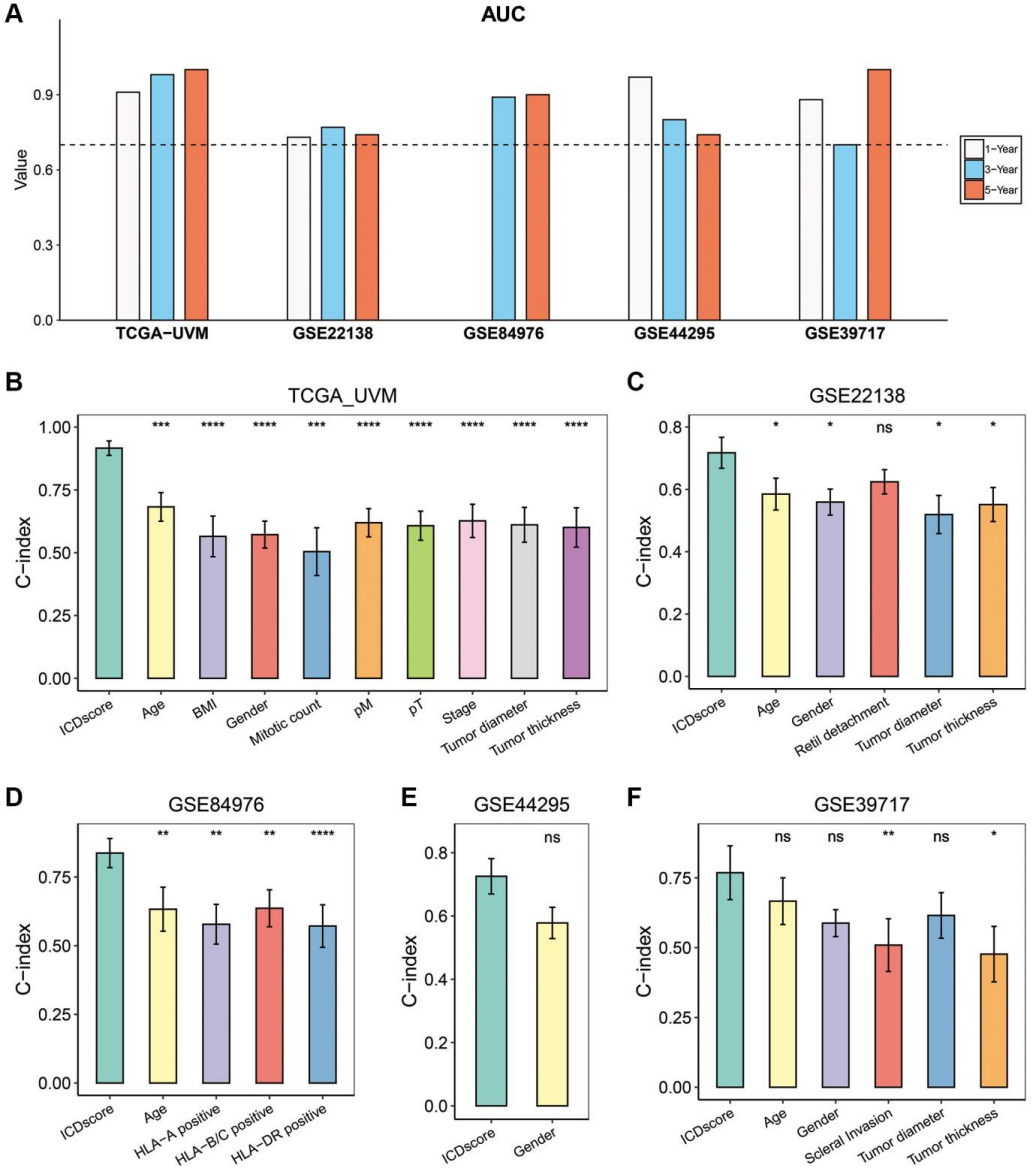
**Evaluation of the ICDscore in UVM.** (**A**) The 1-, 3-, and 5-year AUCs of the ICDscore in the TCGA-UVM, GSE22138, GSE84976, GSE44295, and GSE39717 cohorts. (**B**–**F**) The C-index (95% confidence interval) of the ICDscore and clinical features in the TCGA-UVM (**B**), GSE22138 (**C**), GSE84976 (**D**), GSE44295 (**E**), and GSE39717 (**F**) cohorts. Abbreviation: Ns: not significant. ^*^p < 0.05; ^**^p < 0.01; ^***^p < 0.001; ^****^p < 0.0001.

We first conducted ROC analyses to evaluate the predictability for prognosis of the ICDscore in UVM. As shown in Figure 4A, the 1-, 3-, and 5-year AUCs of the ICDscore were 0.91, 0.98, and 1.00 in the TCGA-UVM cohort; 0.73, 0.77, and 0.74 in the GSE2218 cohort; 0.97, 0.80, and 0.74 in the GSE44295 cohort; 0.88, 0.70, and 1.00 in the GSE39717 cohort. In the GSE84976 cohort, since all patients survived for more than 1 year, the AUC of the ICDscore at 1-year was not measurable, and its 3- and 5-year AUCs were 0.89 and 0.90, respectively (Figure 4A). We also measured and compared the C-index of the ICDscore and other clinical characteristics. As shown in Figure 4B–4F, the C-index [95% confidence interval] of the ICDscore in the five independent cohorts was 0.916 (0.888–0.945) (Figure 4B), 0.717 (0.668–0.767) (Figure 4C), 0.837 (0.784–0.890) (Figure 4D), 0.726 (0.700–0.781) (Figure 4E), and 0.769 (0.672–0.865) (Figure 4F), respectively. In all these cohorts, the C-index of the ICDscore was higher than that of other clinical features such as stage (Figure 4B, *p* < 0.0001), tumor diameter (Figure 4B, 4C and 4F), tumor thickness (Figure 4B, 4C, and 4F). T Huibertus van Esse et al. reported that the expression of human leukocyte antigen (HLA) expression was upregulated in UVM and associated with shorter survival time [33]. As shown in Figure 4D, the C-index of the ICDscore was also higher than that of HLA-A, HLA-B/C, or HLA-DR.

**Figure 5:**
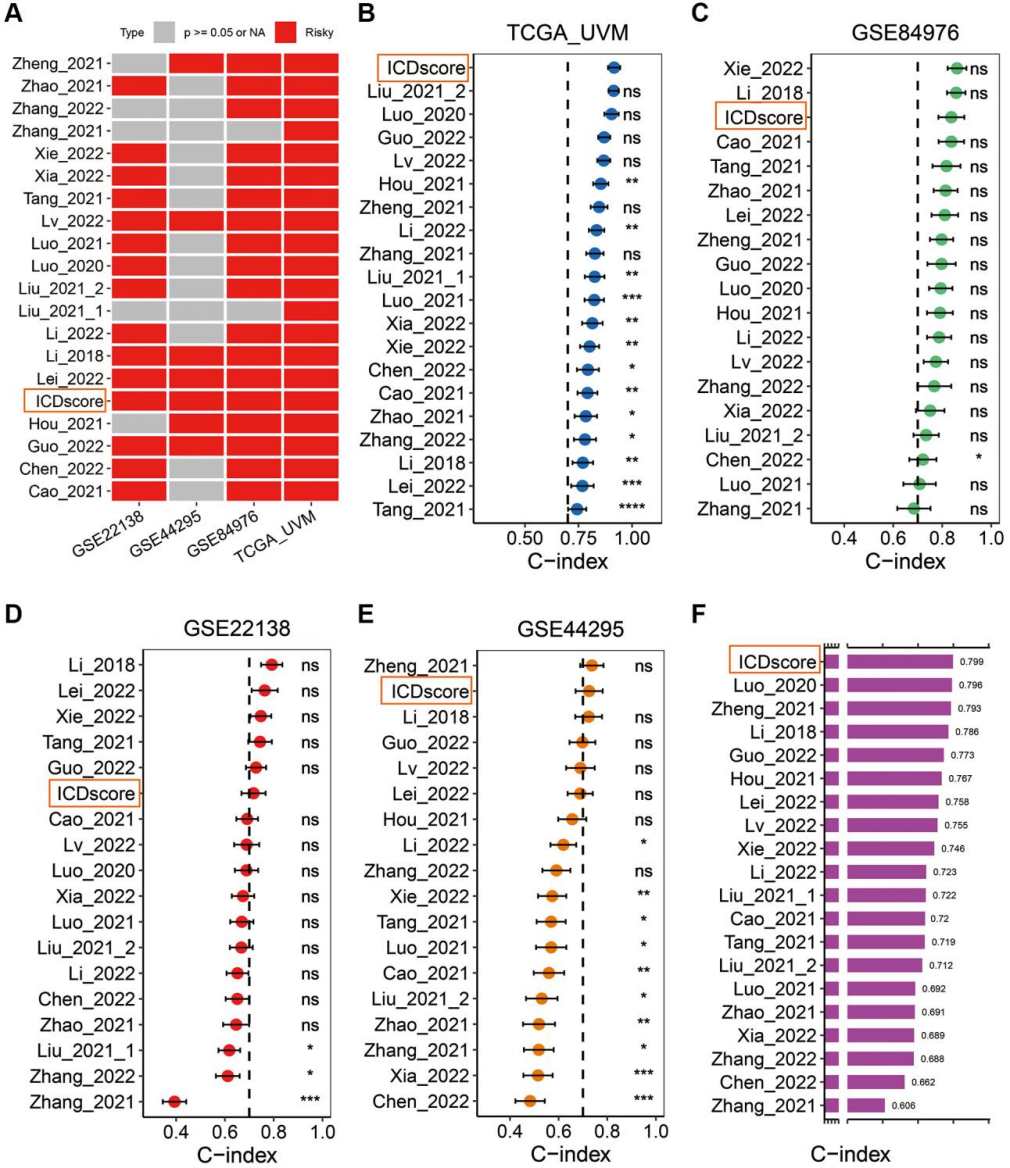
**Comparison between the ICDscore and other published signatures.** (**A**) Univariate Cox regression analysis of the ICDscore and 19 published mRNA signatures in TCGA-UVM, GSE22138, GSE44295 and GSE84976 cohorts. (**B**–**E**) C-index analyses of the ICDscore and 19 published mRNA signatures in TCGA-UVM (**B**), GSE84976 (**C**), GSE22138 (**D**), and GSE44295 (**E**) cohorts. Statistic tests: two-sided z-score test. Data are presented as mean ± 95% confidence interval (CI). (**F**) The average C-index of the ICDscore and 19 published mRNA signatures across all studied cohorts. Abbreviation: Ns: not significant. ^*^p < 0.05; ^**^p < 0.01; ^***^p < 0.001; ^****^p < 0.0001.

In addition, we retrieved mRNA risk models which had been constructed to predict the prognosis of UVM patients by searching the Pubmed website. After excluding some models with no formula available to calculate the risk score [34–38], 19 mRNA signatures (Supplementary Table 3) were finally enrolled to benchmark the ICDscore. These signatures were relevant to many biological processes, including immune cell infiltration [39, 40], autophagy [41], DNA methylation [42], necroptosis [43], pyroptosis [44], cuproptosis [45], ferroptosis [46], epithelial–mesenchymal transition (EMT) [47], and metabolism [48]. Univariate Cox analyses indicated that only the ICDscore and other 4 signatures had significant prognostic relevance across the four cohorts (Figure 5A). The C-index of the ICDscore ranked first, third, sixth and second among all the 20 mRNA signatures in the TCGA-UVM (Figure 5B), GSE84976 (Figure 5C), GSE22138 (Figure 5D) and GSE44295 (Figure 5E) cohorts, and had the highest average C-index (0.799) in all the four cohorts (Figure 5F), suggesting that the ICDscore have a superior performance in prognosis prediction.

### Association between immune characteristics and ICDscore

**Figure 6:**
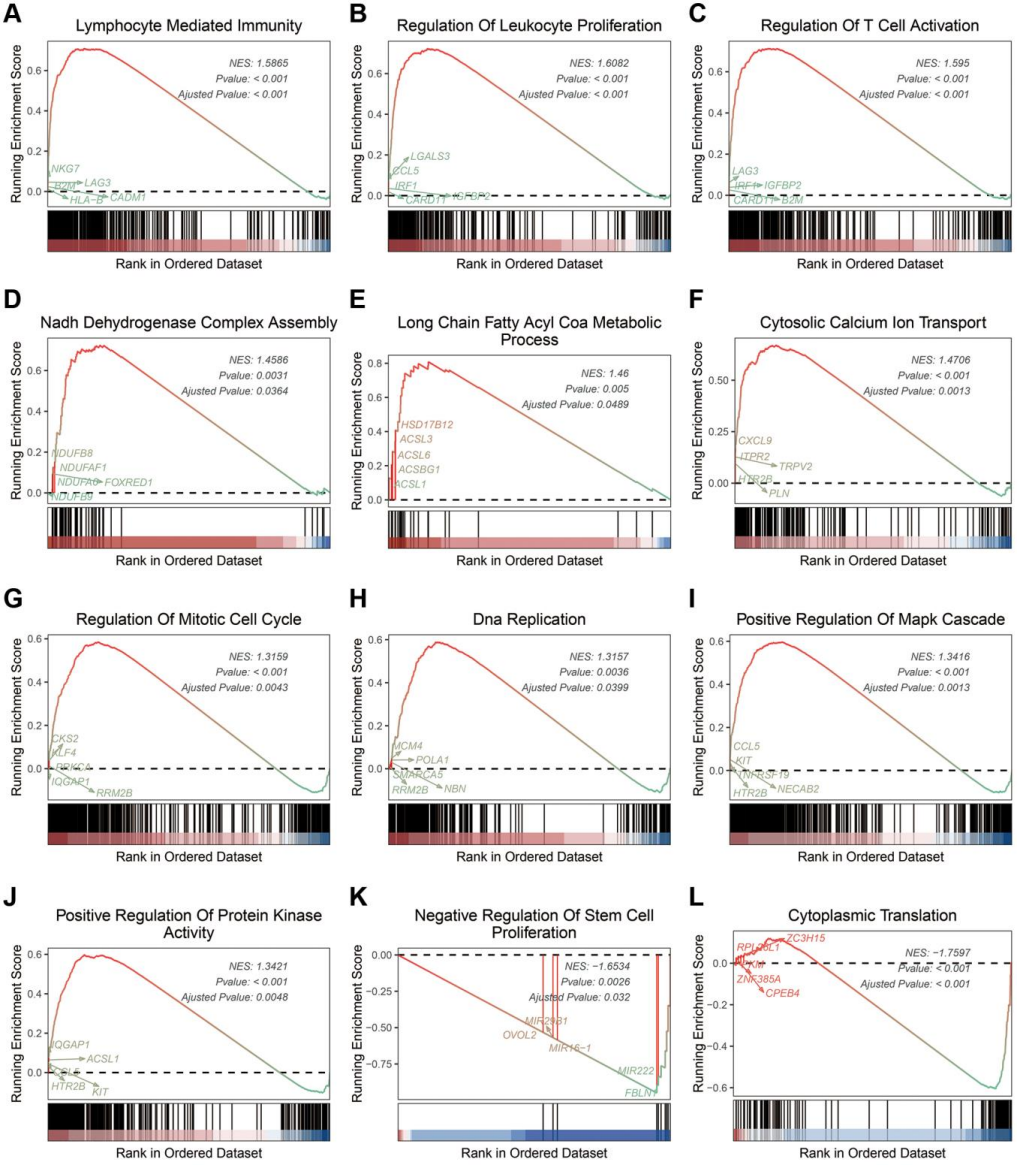
**Biological features of UVM patients in the stratified ICDscore subgroups.** (**A**–**L**) Examples of GSEA results of UVM patients with high-ICDscore (**A**–**J**) or low-ICDscore (**K**, **L**).

To understand the difference between the stratified ICDscore subgroups, GSEA was conducted. UVM patients with high-ICDscore showed significant enrichment in immune related processes (Supplementary Table 4), such as lymphocyte mediated immunity (Figure 6A), regulation of leukocyte proliferation (Figure 6B), and regulation of T cell activation (Figure 6C); in metabolism related processes (Supplementary Table 4), such as NADH dehydrogenase complex assembly (Figure 6D), long chain fatty acid COA metabolic process (Figure 6E), and cytosolic calcium ion transport (Figure 6F); in cell proliferation related pathways (Supplementary Table 4), such as regulation of mitotic cell cycle (Figure 6G), DNA replication (Figure 6H), positive regulation of MAPK cascade (Figure 6I), and positive regulation of protein kinase activity (Figure 6J). Moreover, patients with low-ICDscore were enriched in negative regulation of stem cell proliferation (Figure 6K) and cytoplasmic translation (Figure 6L).

**Figure 7:**
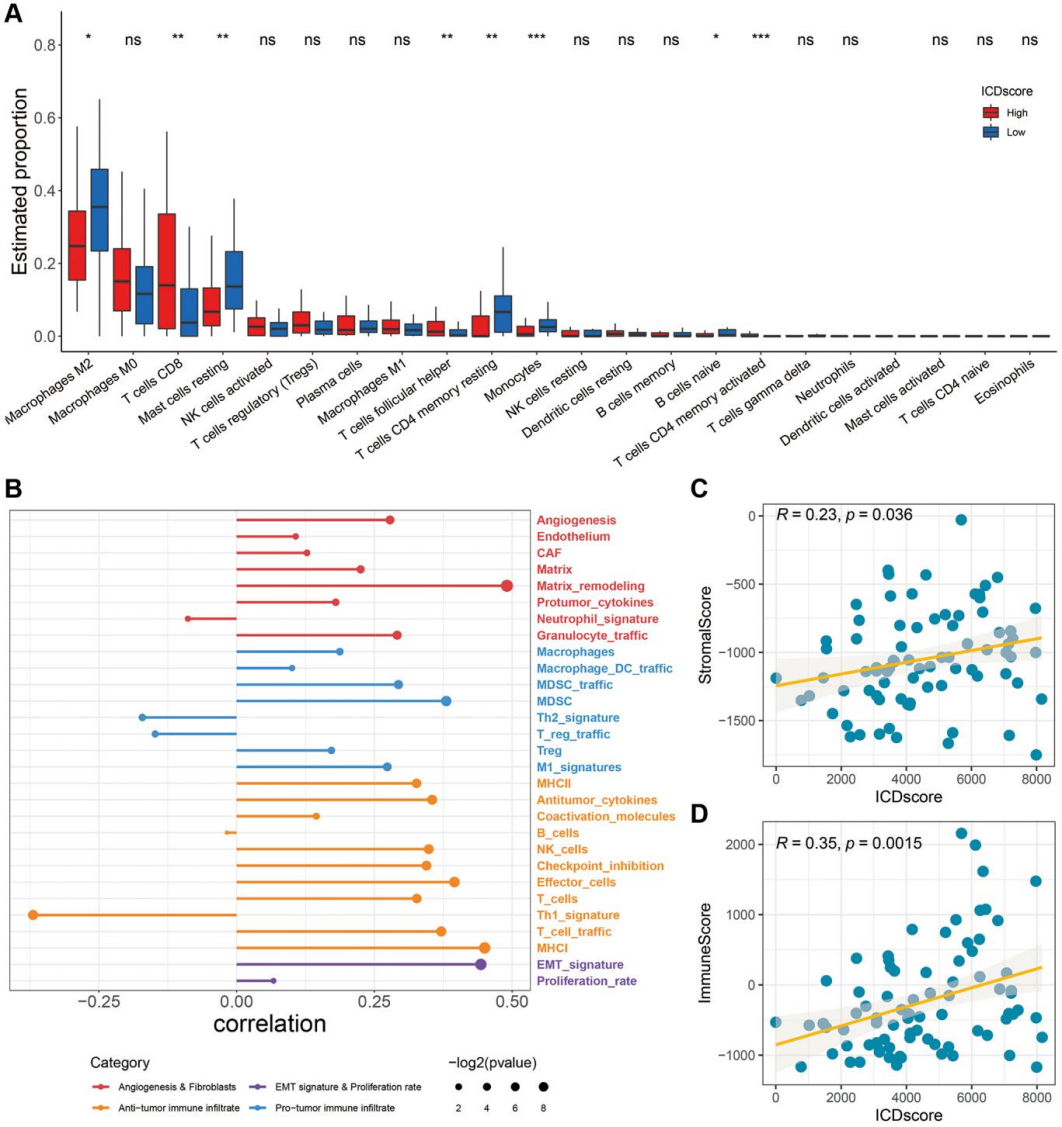
**Association between immune characteristics and the ICDscore.** (**A**) Distribution of 22 types of infiltrating immune cells in patients in the high-ICDscore and low-ICDscore subgroups of the TCGA-UVM cohort. (**B**) Correlation analysis between the ICDscore and 29 Fges. (**C**, **D**) Correlation analysis of the ICDscore with StromalScore (**C**) and ImmuneScore (**D**). Abbreviation: Ns: not significant. ^*^p < 0.05; ^**^p < 0.01; ^***^p < 0.001; ^****^p < 0.0001.

As shown in Figure 7A, patients with high-ICDscore shad higher levels of CD8 T cells and T cells follicular helper, whereas patients with low-ICDscore had higher levels of M2 macrophages, mast cell resting, T cells CD4 memory resting and monocytes (Figure 7A). Recently, Alexander Bagaev et al. characterized the tumor microenvironment (TME) by 29 functional gene expression signatures (Fges), and classified cancer patients into four subtypes [49]. As shown in Figure 7B, the ICDscore had a positive correlation with the expression of most of these Fges. Consistently, the ICDscore also showed a significant positive correlation with StromalScore (Figure 7C, R = 0.23, *p* = 0.036) or ImmuneScore (Figure 7D, R = 0.35, *p* = 0.0015).

### Implication of the ICDscore in anti-tumor therapy

**Figure 8:**
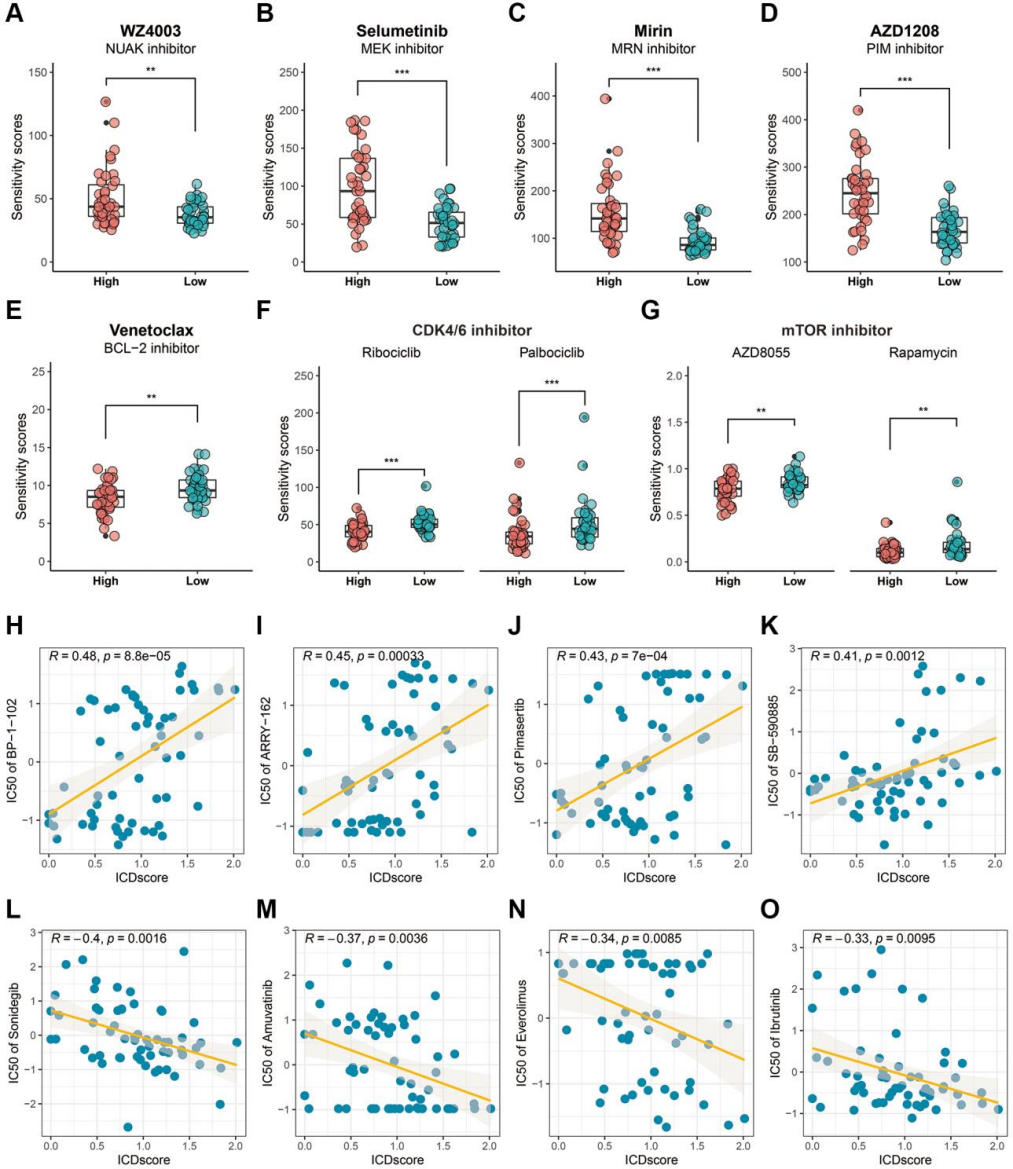
**Drug sensitivity analysis based on the ICDscore grouping.** (**A**–**G**) Sensitivity analysis of WZ4003 (**A**), Selumetinib (**B**), Mirin (**C**), AZD1208 (**D**), Venetoclax (**E**), CDK4/6 inhibitor (**F**), and mTOR inhibitor (**G**) in UVM patients from high-ICDscore and low-ICDscore subgroups. (**H**–**O**) Correlation analysis between the ICDscore and the IC50 of BP-1-102 (**H**), ARRY-162 (**I**), Pimasertib (**J**), SB-590885 (**K**), Sonidegib (**L**), Amuvatinib (**M**), Everolimus (**N**), and Ibrutinib (**O**). Abbreviation: Ns: not significant. ^*^p < 0.05; ^**^p < 0.01; ^***^p < 0.001; ^****^p < 0.0001.

The Genomics of Drug Sensitivity in Cancer (GDSC) and cellMiner databases were used to predict potential anti-tumor drugs for the stratified ICDscore UVM patients. Based on the results from the GDSC database, UVM patients with high-ICDscore exhibited higher sensitivity score to NUAK inhibitor (WZ4003, Figure 8A, *p* < 0.01), MEK inhibitors (Selumetinib, Figure 8B, *p* < 0.001), MRN inhibitor (Mirin, Figure 8C, *p* < 0.001), PIM inhibitor (AZD1208, Figure 8D, *p* < 0.001). UVM patients with low-ICDscore exhibited significantly higher sensitivity score to BCL-2 inhibitor (Venetoclax, Figure 8E, *p* < 0.01), CDK4/6 inhibitors (Ribociclib and Palbociclib, Figure 8F, *p* < 0.001), and mTOR inhibitors (AZD8055 and Rapamycin, Figure 8G, *p* < 0.01). The cellMiner database results indicated that the ICDscore showed a significantly positive correlation with STAT3 inhibitor (BP-1-102, Figure 8H, R = 0.48, *p* = 8.8e-05); with many MEK inhibitors, such as ARRY-162 (Figure 8I, R = 0.45, *p* = 0.00033), Pimasertib (Figure 8J, R = 0.43, *p* = 7e-04); and with B-RAF inhibitor (SB-590885, Figure 8K, R = 0.41, *p* = 0.0012), and a significantly negative correlation with SMO inhibitor (Sonidegib, Figure 8L, R = −0.4, *p* = 0.0016); with Amuvatinib (a multi-kinase inhibitor, Figure 8M, R = −0.37, *p* = 0.0038); with Everolimus (mTOR inhibitor, Figure 8N, R = −0.34, *p* = 0.0085); and with Ibrutinib (BTK inhibitor, Figure 8O, R = −0.33, *p* = 0.0095).

**Figure 9:**
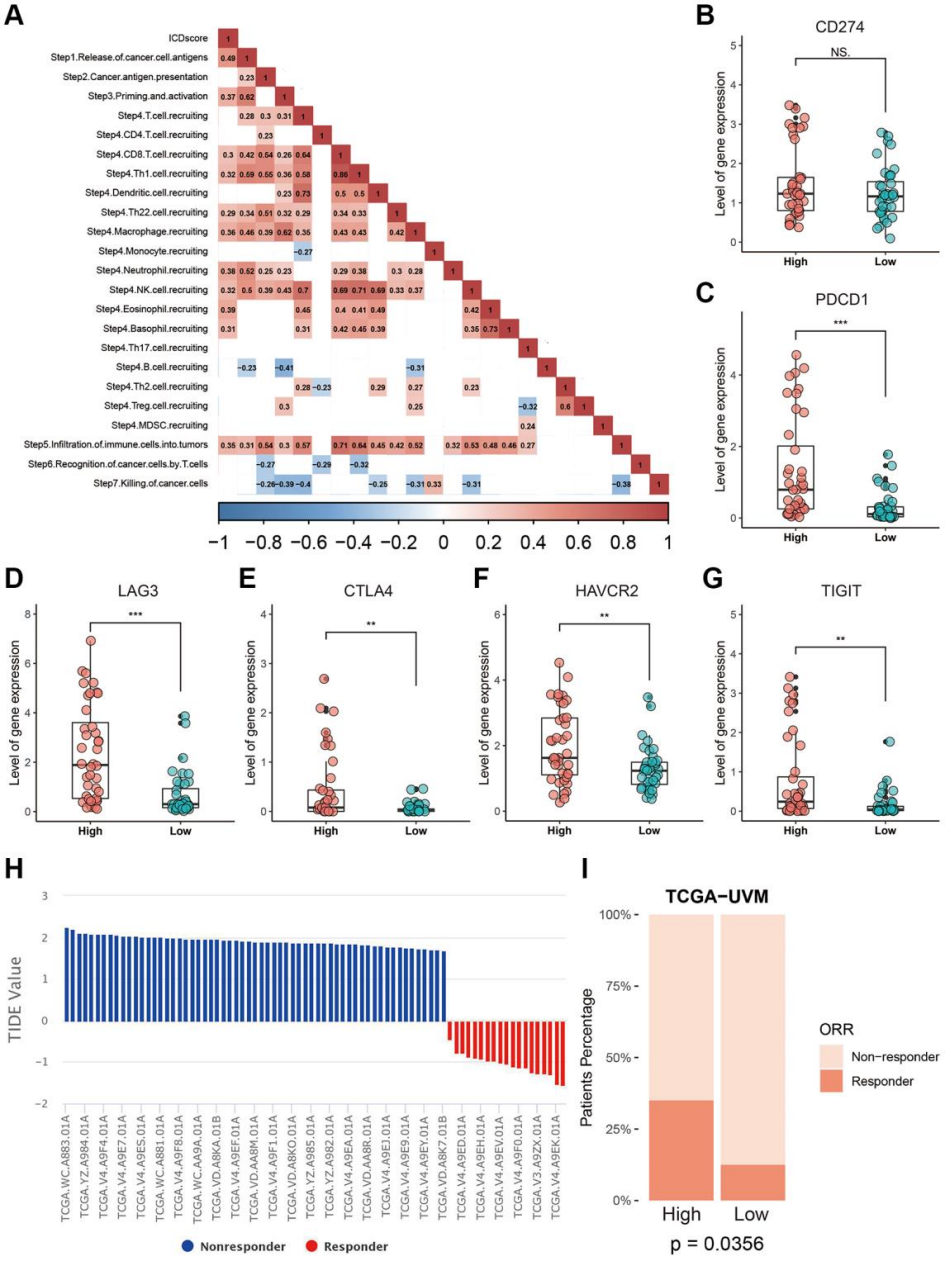
**Correlation between the ICDscore and anti-tumor immunotherapy.** (**A**) Correlation analysis between the ICDscore and the status of anti-cancer immunity. (**B**–**G**) The expression levels of CD274, PDCD1, LAG3, CTLA4, HAVCR2, and TIGIT in UVM patients in the high-ICDscore and the low-ICDscore subgroups of the TCGA-UVM cohort. (**H**) TIDE score and immunotherapy response rate of UVM patients from TCGA-UVM cohort. (**I**) Immunotherapy response rate of UVM patients from TCGA-UVM cohort with high-ICDscore and low-ICDscore. Abbreviation: Ns: not significant. ^*^p < 0.05; ^**^p < 0.01; ^***^p < 0.001.

The potential application of the ICDscore in immunotherapy was also investigated, since UVM patients with high-ICDscore were enriched in immune related processes (Figure 6A–6C) and exhibited significantly higher level of CD8 T cells (Figure 7A). We analyzed the status of anti-cancer immunity by downloading data from the Tracking Tumor Immunophenotype (TIP) database. As shown in Figure 9A, the ICDscore showed significantly positive correlation with release of cancer cell antigens (step 1), priming and activation (step 3), recruiting of many immune cells such as CD8 T cells and neutrophils (step 4), and infiltration of immune cells into tumors (step 5). Although the expression of CD274 was not different between the high- and low-ICDscore subgroups (Figure 9B), the expression of several other immune checkpoint inhibitors including PDCD1 (Figure 9C, *p* < 0.001), LAG3 (Figure 9D, *p* < 0.001), CTLA4 (Figure 9E, *p* < 0.01), HAVCR2 (Figure 9F, *p* < 0.01), and TIGIT (Figure 9G, *p* < 0.01), were all significantly higher in UVM patients with high-ICDscore. Finally, we uploaded the normalized RNA-seq data into the TIDE website and calculated the TIDE score for each sample in TCGA-UVM (Figure 9H). 23.75% of patients in the TCGA-UVM were predicted to be responders to immunotherapy (Supplementary Table 5). In particular, we noticed that patients in the high-ICDscore subgroup were predicted to have a higher percentage of patients responding to immunotherapy (Figure 9I, *p* = 0.0356).

### Role of PARP8 in UVM

**Figure 10:**
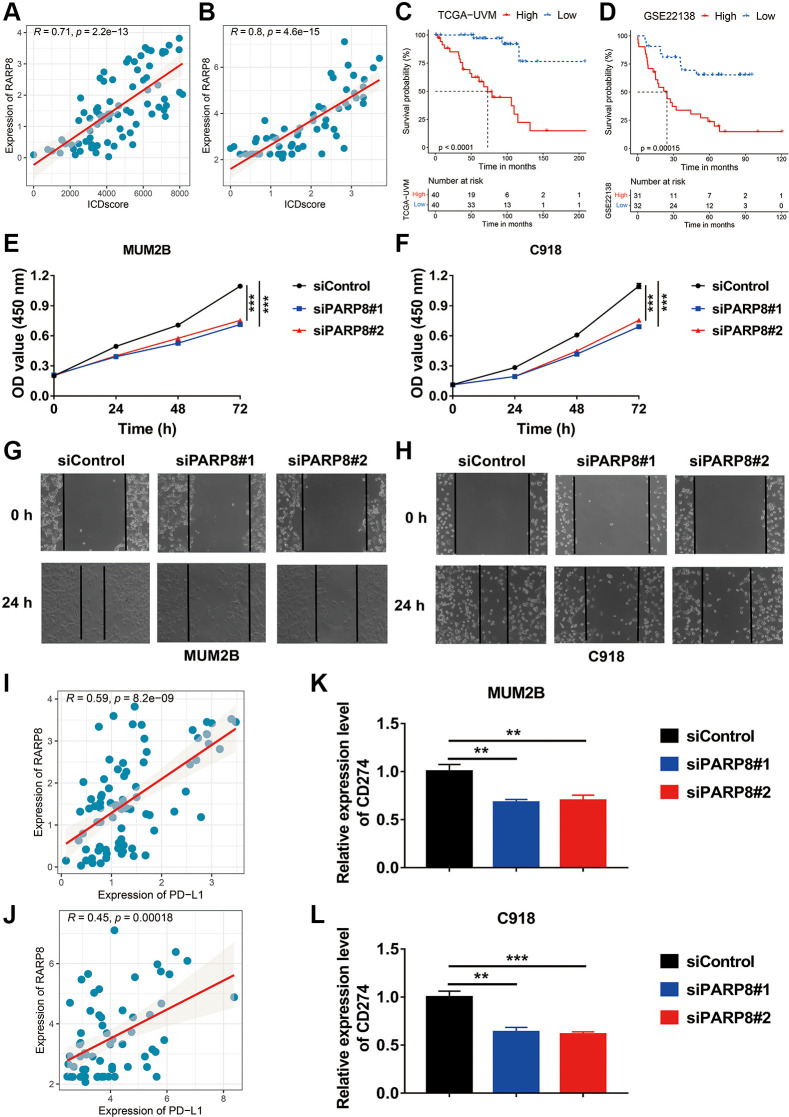
**Role of PARP8 in uveal melanoma cells.** (**A**, **B**) The expression level of PARP8 was positively correlated with the ICDscore in patients from TCGA-UVM (**A**) and GSE22138 (**B**) cohorts. (**C**, **D**) Kaplan-Meier analysis showed that patients with high PARP8 expression had a longer OS in TCGA-UVM (**C**) and GSE22138 (**D**) cohorts. (**E**, **F**) CCK-8 assay showed that PARP8 knockdown in MUB2B or C918 cell inhibited cell proliferation. (**G**, **H**) CCK-8 assay showed that PARP8 knockout in MUB2B and C918 inhibited cell proliferation. (**I**, **J**) In TCGA-UVM and GSEGSE22138 cohorts, PARP8 was positively correlated with CD274 expression. (**K**, **L**) qPCR assay showed that PARP8 knockout in MUB2B and C918 could inhibit CD274 expression. Abbreviation: Ns: not significant. ^*^p < 0.05; ^**^p < 0.01; ^***^p < 0.001.

Among the five key genes used to develop the ICDscore, PARP8 and PRKCDBP had a significant positive correlation with the ICDscore (Figure 2D, Figure 10A, 10B). UVM patients with high expression of PARP8 or PRKCDBP also exhibited shorter OS (Figure 10C, 10D, Supplementary Figure 1A, 1B). To reveal the role of PARP8 in UVM, we knocked down the expression of PARP8 in two UVM cell lines (Supplementary Figure 1C, 1D). CCK8 assay results suggested that downregulation of PARP8 caused decreased cell proliferation of UVM cells (Figure 10E, 10F). Besides, UVM cells with decreased expression of PARP8 showed a slower migration rate than the control groups (Figure 10G, 10H). To further examine the role of PARP8 in tumor microenvironment, we analyzed the relationship between PARP8 and the expression of immune inhibitors. Since the expression of PDCD1, LAG3, CTLA4, HAVCR2, and TIGIT was extremely low in both MUM2B and C918 cells, we focused on the expression of CD274. As shown in Figure 10I, 10J, the ICDscore had a significantly positive correlation with the expression of CD274. Moreover, knockdown of PARP8 in both UVM cells led to decreased expression of CD274, suggesting PARP8 might contribute the expression of the latter gene (Figure 10K, 10L).

## DISCUSSION

Immune checkpoint inhibitors have brought extraordinary benefits to patients with several types of cancer [1, 2, 4]. However, according to some preliminary clinical trials, UVM patients showed no or low overall response rate (ORR) to PD-1 and PD-L1 antibodies [50–52]. Is UVM an exception in the era of immune therapy? The answer might be no. In a recently published phase II study, Meredith S Pelster et al. reported that 18% of UVM patients showed response to nivolumab with ipilimumab, including one complete response and five partial responses. The median progression-free survival (PFS) and OS reached 5.5 months and 19.1 months, respectively [53]. Smita S Chandran et al. reported that 35% of UVM patients achieved objective tumor regression to adoptive T-cell therapy in a single-center, single-arm, and phase II study [54]. Although a better understanding of the immune-escape mechanisms of UVM might be translated into improved ORR in the future [50], identifying useful biomarker could be an alternative and applicable method in selecting UVM patients benefiting from immunotherapy.

ICD is unique in its ability to elicit adaptive immunity, providing the potential to convert a ‘cold’ tumor into a ‘hot’ one [55]. However, the immunogenicity of tumor cells exposed to ICD-inducer is lost in mice presenting with genetic defects in TLR4 or MYD88, suggesting not all tumor cells will ultimately elicit an antitumor-specific T-cell immunity in the presence of ICD-inducer such as chemotherapy or radiotherapy [56]. In this work, we used the ICD-related genes to develop a biomarker to indicate prognosis and immunotherapy sensitivity of UVM patients. We found that based on the expression of ICD-related genes in two independent cohorts, UVM patients could be divided in two clusters with distinct prognosis (Figure 1A–1F). Further, we integrated a set of bioinformatics tools to develop an ICD-related signature, the ICDscore, which might be applicable in the clinics (Figure 2A–2D). In the training cohort (TCGA-UVM) and four independent validating cohorts (GSE22138, GSE84976, GSE44295, and GSE39717), UVM patients with high-ICDscore showed longer survival time (Figure 2E–2I). Although age, tumor size (including diameter and thickness), gender, TNM stage, and other clinical features are associated with survival time of UVM patients [57, 58], the ICDscore exhibited a strong capability in predicting prognosis (Figure 4A) and showed superior performance than the above-mentioned clinical parameters (Figure 4B–4F). In addition, the ICDscore was also compared with 19 previously published mRNA risk models (Figure 5A–5F). These signatures had a tight relevance with a number of biological processes, such as immune cell infiltration [39, 40], various forms of cell death [41, 43, 44], DNA methylation [42], epithelial–mesenchymal transition (EMT) [47], and metabolism [48]. Univariate Cox regression showed that only the ICDscore and four other signatures exhibited prognostic significance across all studied cohorts [47, 59–61], indicating most signatures had a weak association with prognosis or had not been thoroughly validated (Figure 5A). Likewise, the ICDscore had stable performance across multiple cohorts and its average C-index was the highest (Figure 5B–5F), exhibiting an advantage in predicting prognosis of UVM patients.

ICD-related mRNA signatures have also been developed in other types of cancer [25, 26, 62, 63]. Based on results in this work (Figures 2 and 9), Jiayang Cai’s study [62], and Zhiqiang Sun’s study, UVM and glioma patients in the ICD-high risk groups were all associated with poor prognosis. However, patients with head and neck squamous cell carcinoma (HNSCC) in the ICD-high risk groups were associated with longer survival [25]. Despite this, ICD- related risk scores were all associated with high activity of immune response signaling and abundant immune cell infiltration in these cancer types. These patients in the ICD-high risk group were more likely benefit from immunotherapy [25, 62]. The difference in the association between ICD-related risk score and prognosis in different types of cancer might be due to the tumor microenvironment (TME) (REF). Many studies have revealed that a high proportion of CD8 T cells was associated with poor prognosis in patients with UVM and glioma [64, 65], suggesting an immunosuppressive TME and T cell exhaustion in both types of cancer [66, 67].

PARP8, a crucial gene identified in this work to develop the ICDscore, involves in protein auto-ADP-ribosylation and protein mono-ADP-ribosylation [68]. Although the exact function of PARP8 in UVM is unclear, it might regulate different cellular processes, such signal transduction, cell cycle regulation, DNA repair and apoptosis [69]. Based on our *in vitro* experiments, PARP8 might function as an oncogene since its downregulation impairs proliferation and migration of UVM cells (Figure 10E–10H). PARP8 might also contribute to the immunosuppressive status of TME in UVM since its expression had a strong positive correlation with CD274, a well-known immune checkpoint [70]. In addition, silencing the expression of PARP8 caused a downregulation of CD274. PARP1, another member of the PARP family, has been found to play a key role in the immune modulation of tumors, and the inhibition of PARP1 is able to induce innate immunity [71]. Taken together, PARP8 is likely to regulate innate immunity and occurrence of ICD, but more work is needed to demonstrate such a correlation.

## CONCLUSIONS

In conclusion, we a robust ICD-related signature for evaluating the prognosis and benefits of immunotherapy of UVM patients. The ICDscore was superior than other mRNA signatures and served as a promising tool to guide decision-making and surveillance for UVM patients.

## Supplementary Materials

Supplementary Figure 1

Supplementary Table 1

Supplementary Table 2-5
