# Supplementary material for: Identification and validation of immunogenic cell death-related score in uveal melanoma to improve prediction of prognosis and response to immunotherapy
Source: Aging (Albany NY). 2023 May 3;15(9):3442–64. doi: 10.18632/aging.204680 (PMC10449274; doi:10.18632/aging.204680)
Supplement: Supplementary Figure 1 [file aging-15-204680-s001.pdf]

SUPPLEMENTARY FIGURE

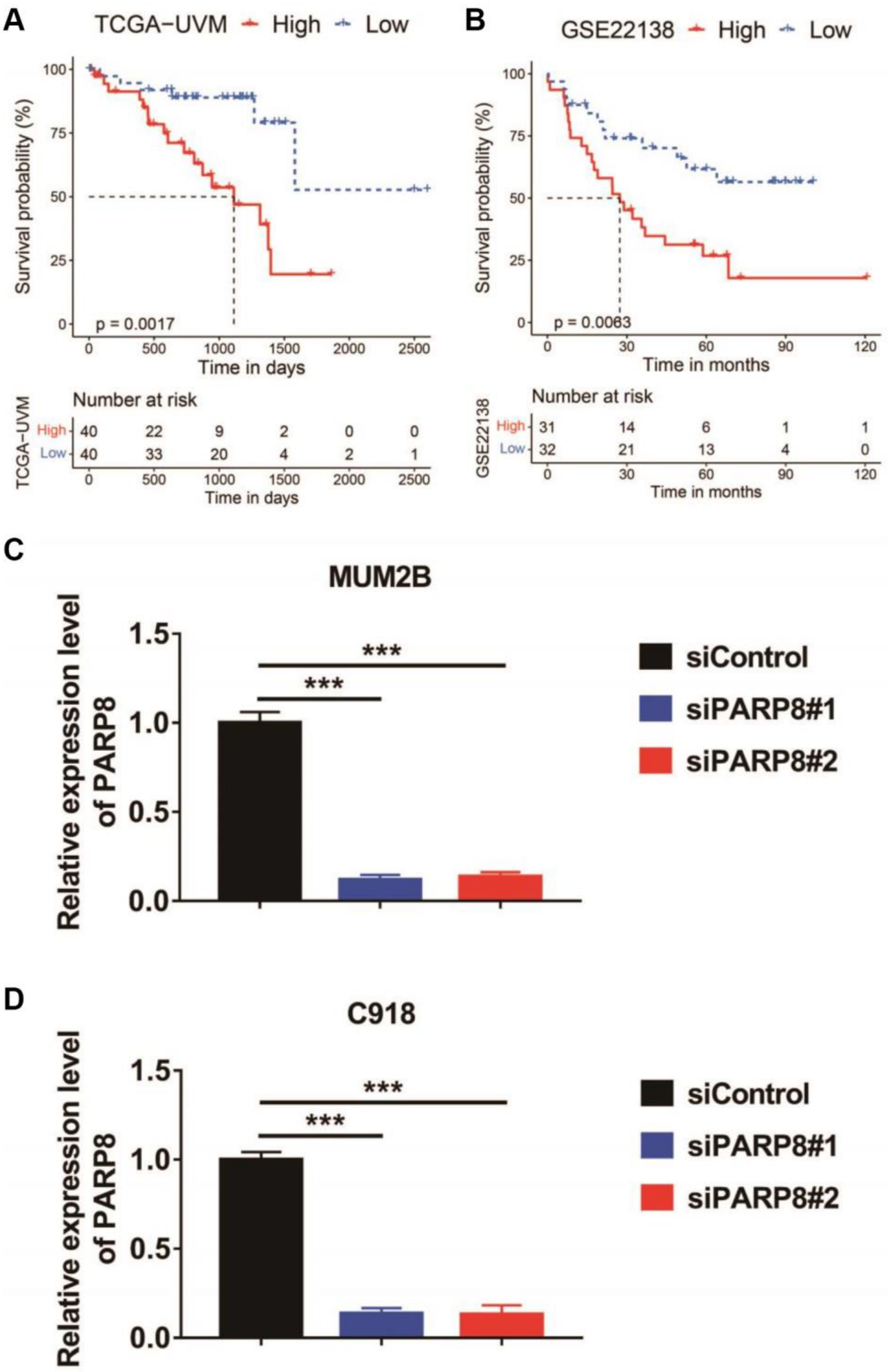

Supplementary Figure 1. (**A**, **B**) Kaplan–Meier curves of OS in the PRKCDBP-high and PRKCDBP-low subgroups of UVM patients from the TCGA-UVM (**A**) and GSE22138 (**B**) cohorts. (**C**, **D**) qPCR result of PARP8 in MUM2B (**C**) and C918 (**D**) cells transfected with siPARP8 or negative control.
