# Supplementary material for: Identification and validation of immunogenic cell death-related score in uveal melanoma to improve prediction of prognosis and response to immunotherapy
Source: Aging (Albany NY). 2023 May 3;15(9):3442–64. doi: 10.18632/aging.204680 (PMC10449274; doi:10.18632/aging.204680)
Supplement: Supplementary Table 1 [file aging-15-204680-s002.pdf]

SUPPLEMENTARY TABLE

Supplementary Table 1. The sequences of siRNA and qPCR primer.

Application		Forward sequence	Reverse sequence
PARP8#1	qPCR	5′-TACGGAGGGCAGGTGAACTA-3′	5′-TTCCCAAGCCACAGCAATCT-3′
PARP8#2	qPCR	5′-TACGGAGGGCAGGTGAACTA-3′	5′-TTCCCAAGCCACAGCAATCT-3′
CD274#1	qPCR	5′-CTGGCATTTGCTGAACGCAT-3′	5′-AGGTCTTCCTCTCCATGCAC-3′
CD274#2	qPCR	5′-CTGGCATTTGCTGAACGCAT-3′	5′-AGGTCTTCCTCTCCATGCAC-3′
GAPDH	qPCR	5′-TCGGAGTCAACGGATTTGGT-3′	5′-TCGCCCCACTTGATTTTGGA-3′
PARP8#1	siRNA	5′-GGUGGUAGAUCUACUAGUAUC-3′	5′-UACUAGUAGAUCUACCACCUG-3′
PARP8#2	siRNA	5′-CGACUACACUGUUCACUUACA-3′	5′-UAAGUGAACAGUGUAGUCGAA-3′
Negative control	siRNA	5′-UUCUCCGAACGUGUCACGUTT-3′	5′-ACGUGACACGUUCGGAGAATT-3′
